# RETRACTION: Effect of Enteral Immunonutrition Compared With Enteral Nutrition on Surgical Wound Infection, Immune and inflammatory Factors, Serum Proteins, and Cellular Immunity in Subjects With Gastric Cancer Undergoing a Total Gastrectomy: A Meta‐Analysis

**DOI:** 10.1111/iwj.70343

**Published:** 2025-03-17

## Abstract

**RETRACTION:** FuH. , LiB. , and LiangZ. , “Effect of Enteral Immunonutrition Compared With Enteral Nutrition on Surgical Wound Infection, Immune and inflammatory Factors, Serum Proteins, and Cellular Immunity in Subjects With Gastric Cancer Undergoing a Total Gastrectomy: A Meta‐Analysis,” International Wound Journal 19, no. 7 (2022): 1625–1636, 10.1111/iwj.13763.35352476 PMC9615293

The above article, published online on 29 March 2022, in Wiley Online Library (http://onlinelibrary.wiley.com/), has been retracted by agreement between the journal Editor in Chief, Professor Keith Harding; and John Wiley & Sons Ltd. Following an investigation by the publisher, all parties have concluded that this article was accepted solely on the basis of a compromised peer review process. The editors have therefore decided to retract the article. The authors did not respond to our notice regarding the retraction.

**RETRACTION:** H. Fu , B. Li , and Z. Liang , “Effect of Enteral Immunonutrition Compared With Enteral Nutrition on Surgical Wound Infection, Immune and inflammatory Factors, Serum Proteins, and Cellular Immunity in Subjects With Gastric Cancer Undergoing a Total Gastrectomy: A Meta‐Analysis,” International Wound Journal 19, no. 7 (2022): 1625–1636, 10.1111/iwj.13763.35352476 PMC9615293

The above article, published online on 29 March 2022, in Wiley Online Library (http://onlinelibrary.wiley.com/), has been retracted by agreement between the journal Editor in Chief, Professor Keith Harding; and John Wiley & Sons Ltd. Following an investigation by the publisher, all parties have concluded that this article was accepted solely on the basis of a compromised peer review process. The editors have therefore decided to retract the article. The authors did not respond to our notice regarding the retraction.

